# Cytokine profiling in active and quiescent SLE reveals distinct patient subpopulations

**DOI:** 10.1186/s13075-018-1666-0

**Published:** 2018-08-09

**Authors:** John A. Reynolds, Eoghan M. McCarthy, Sahena Haque, Pintip Ngamjanyaporn, Jamie C. Sergeant, Elaine Lee, Eileen Lee, Stephen A. Kilfeather, Ben Parker, Ian N. Bruce

**Affiliations:** 1 Arthritis Research UK Centre for Epidemiology, Centre for Musculoskeletal Research, Manchester Academic Health Science Centre, University of Manchester, Manchester, M13 9PT UK; 2 The Kellgren Centre for Rheumatology, NIHR Manchester Biomedical Research Centre, Manchester University Hospitals NHS Foundation Trust, Manchester Academic Health Science Centre, Manchester, UK; 3 Rheumatology Department, Wythenshawe Hospital, Manchester University Hospitals NHS Foundation, Manchester, UK; 4 Division of Allergy, Immunology and Rheumatology, Department of Internal Medcine, Faculty of Medicine, Ramathibodi Hospital, Mahidol University, Bangkok, Thailand; 5 Centre for Biostatistics, Manchester Academic Health Science Centre, University of Manchester, Manchester, UK; 6 Aeirtec Ltd, The SmokeHouses Building, Clifford Fort, North Shields, Newcastle upon Tyne, UK

**Keywords:** Systemic lupus erythematosus, Cytokines, Biomarkers, Disease activity, Cluster analysis

## Abstract

**Background:**

Patients with SLE display marked clinical and immunlogical heterogeneity. The purpose of the study was to investigate patterns of serum cytokines in patients with active and stable systemic lupus erythematosus (SLE) and to determine how they relate to clinical phenotype.

**Methods:**

Serum levels of 10 cytokines were measured retrospectively in a cohort of patients with SLE and in healthy controls using a high-sensitivity multiplex bead array. Disease activity was determined using the Systemic Lupus Erythematosus Disease Activity Index 2000 (SLEDAI-2K) and British Isles Lupus Assessment Group (BILAG-2004) indices. Logistic regression models were used to determine the association between cytokine levels and active SLE. Principal component analysis (PCA) and cluster analysis was then used to identify subgroups of patients on the basis of cytokine levels.

**Results:**

Serum chemokine (C-X-C motif) ligand 10 (CXCL10) and CXCL13 were significantly higher in patients with SLE compared to healthy controls. Two cytokines (pentraxin-related protein (PTX3) and CXCL10) were significantly higher in patients with active disease after adjustment for potential confounding factors. Measurement of four cytokines (CXCL10, IL-10, IL-21 and PTX3) significantly improved the performance of a model to identify patients with clinically active disease. Cluster analysis revealed that the patients formed 3 distinct groups, characterised by higher levels of interferon alpha (IFNα) and B lymphocyte stimulator (BLyS) (group 1), increased CXCL10 and CXCL13 (group 2) or low levels of cytokines (group 3). Group 2 had significantly lower serum complement and higher anti-double-stranded DNA antibodies and increased prevalence of inflammatory arthritis.

**Conclusions:**

Multiplex analysis has identified a serum cytokine signature for active SLE. Within the SLE population distinct cytokine subgroups were identified, with differing clinical and immunological phenotypes that appeared stable over time. Assessment of cytokine profiles may reveal unique insights into disease heterogeneity.

**Electronic supplementary material:**

The online version of this article (10.1186/s13075-018-1666-0) contains supplementary material, which is available to authorized users.

## Background

Systemic lupus erythematosus (SLE) is a systemic inflammatory autoimmune disease with a broad clinical and immunological phenotype, and marked variability in response to treatment. The ability to understand this heterogeneity and to develop relevant biomarkers, in order to better direct therapeutic decision-making, is an important unmet need in the care of patients with lupus [1].

The observed clinical heterogeneity likely reflects differences in underlying immunopathological processes. Recently a landmark study by Banchereau et al. used whole blood transcriptional profiling to identify seven transcriptionally distinct groups in a paediatric SLE population [2]. A number of studies have also identified autoantibody clusters in SLE, again reflecting the immunopathological heterogeneity within this patient group [3, 4].

Although a number of genetic, epigenetic and protein biomarkers have been proposed, many of these currently have limited clinical utility [5]. Cytokine levels may be influenced by a number of endogenous and exogenous factors including clinical disease activity, circadian variation, the presence of infection and immunosuppressant treatment [6]. Although patients with SLE are reported to have increased levels of many cytokines including interleukin (IL)-10, IL-17 and interferon alpha (IFNα) these are often measured in isolation making it difficult to understand how cytokine networks exist in SLE [7].

A number of cytokines have been implicated in the pathogenesis of SLE (reviewed by Yu et al. 2012 [8]). However there are currently no reliable cytokine biomarkers to differentiate between active and inactive SLE. Biomarkers that can assist in the identification of active disease are desirable as the clinical assessment of patients can be difficult due to the accrual of damage and the presence of other comorbidities (including concurrent infection).

Furthermore, it is possible that some cytokines are more strongly related to a specific disease phenotype rather than disease activity. As an example, IL-17 has been associated with central nervous system disease in patients with SLE independently of disease activity [9]. This suggests that cytokine levels may reveal important additional information about the immunophenotype of patients with SLE. In addition, although it is recognised that cytokines are unlikely to function in isolation from one another, relatively little is known about how individual cytokines relate to each other in the disease state.

To date, the majority of studies have used singleplex plate-based enzyme-linked immunosorbent assays (ELISAs) to detect serum or plasma cytokine levels. These methods may have disadvantages including a narrow dynamic range, ability to measure only a single analyte and in some cases relatively low sensitivity. Initial multiplexing systems have relatively low sensitivity and have been used by many as “screening systems” prior to further exploration of observed trends with singleplex assays. The sensitivity of multiplexing, however, has evolved to match singleplex assays and, in certain areas, surpasses single ELISA assay sensitivity.

The aim of the present study was to use a high-sensitivity multiplex bead-based assay to investigate serum cytokine levels in patients with stable and active SLE. We incorporated the highest-sensitivity assay system available to us in a customised multiplex system to provide simultaneous cytokine measurement, in order to better understand cytokine relationships in SLE. Specifically we aimed to determine how these cytokines related to disease activity and to determine whether individual cytokines or groups of cytokines were associated with specific SLE disease features.

## Methods

Patients with SLE (scoring ≥ 4 in the 1997 updated American College of Rheumatology (ACR) classification criteria [10]) were recruited from Manchester University Hospitals National Health Service (NHS) Foundation Trust between 2007 and 2013. All patients provided informed written consent, and the study was approved by UK Research Ethics Committees. Demographic details including ethnicity and smoking status were recorded, patients’ disease activity and concurrent medication was assessed by a physician and fasting blood samples were taken. Clinical features and serological data relevant to the ACR classification criteria were collected retrospectively from hospital records. Serum autoantibodies and complement levels were measured using standard laboratory assays. In a subset of patients with clinically stable SLE, repeat samples were obtained after 3 months.

### Measurement of disease activity

SLE disease activity was measured using the Systemic Lupus Disease Activity Index 2000 (SLEDAI-2 K) [11] and the British Isles Lupus Assessment Group 2004 (BILAG-2004) [12]. Active disease was defined as (i) SLEDAI-2 K > 4, and/or (ii) one BILAG-2004 “A” score and/or (iii) two BILAG-2004 “B” scores.

### Cytokine measurement

Serum samples were stored at − 80 °C until analysis. We performed a literature review of studies of the immunopathogenesis of SLE and we selected a panel of 10 cytokines to cover the principle pathways deemed to be relevant to the pathogenesis of SLE: IFNα, B lymphocyte stimulator (BLyS), IL-10, IL-17, IL-18, IL-21, C-X-C motif chemokine ligand (CXCL10), CXCL13, monocyte chemotactic protein-1 (MCP-1) and pentraxin related protein 3 (PTX3). These cytokines therefore addressed monocytes/innate cells (IFNα, IL-10, IL-18, CXCL10, MCP-1 and PTX3), B cells (BLyS and CXCL13) and T cells (IL-10, IL-17 and IL-21). Serum cytokine levels were measured using a high-sensitivity bead-based multiplex ELISA (Aeirtec Ltd., UK). Limits of detection (LOD) values in picogram/millilitre determined within the analysis were as follows: BLyS, 0.05; CXCL13, 0.03; IFNα, 0.11; IL-10, 0.18; IL-17, 0.07; IL-18, 0.64; IL-21, 0.06; CXCL10, 0.05; MCP-1, 0.15 and PTX3, 1.43.

### Statistical methods

Cytokine levels were compared between groups using non-parametric tests. Logistic regression models were used to compare patients with SLE and healthy controls and patient groups with active and inactive disease. In order to investigate which cytokines were most associated with the presence of active disease, multivariable models were developed (using a modified definition of active disease, which excluded the contribution of low serum complement/high anti-double-stranded (ds)DNA to the SLEDAI score) and adjusted for age, ethnicity, disease duration and serological markers. Non-significant predictors were removed using backwards elimination (probability threshold for removal 0.1). The ability of the model to discriminate between patients with active and inactive disease was measured using the area under the receiver operating characteristic curve (AUC).

To investigate networks, a Spearman correlation matrix was constructed. For the cluster analysis, principal component analysis (PCA) was undertaken using the cytokine data alone to determine the number of groups present. Following this, K-means clustering of the standardised cytokine levels was conducted. The clinical and serological features of patients were then compared among the three cytokine-based groups. Statistical analysis was carried out using GraphPad Prism v7.0 (GraphPad Software, Inc), STATA/MP v13.0 (StataCorp LLC) and R v3.3.2.

## Results

**Fig. 1:**
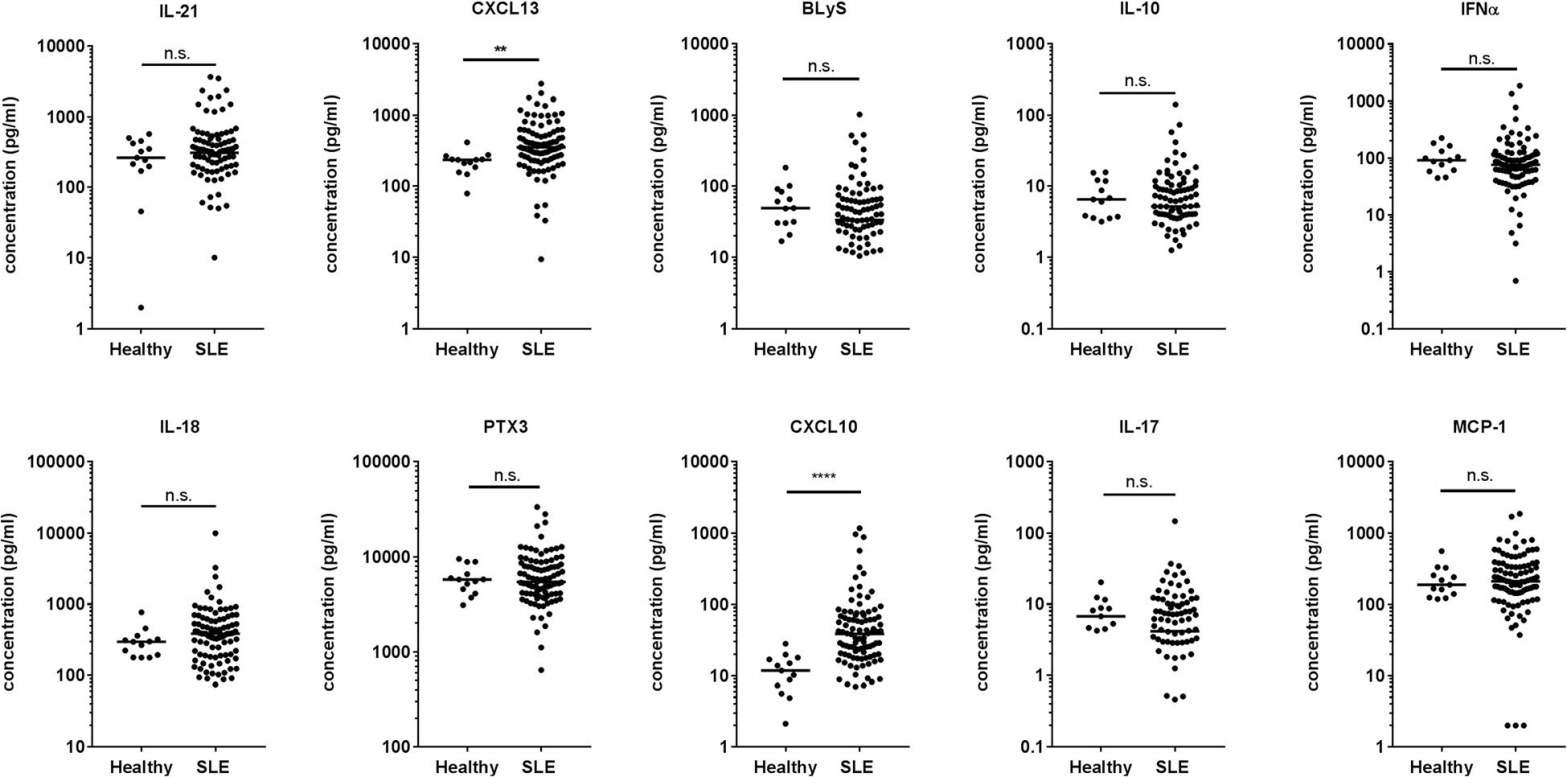
Differences in cytokine levels between patients with systemic lupus erythematosus (SLE) and healthy subjects. The plots show the serum level (picograms/millilitre) of each of the 10 cytokines in patients with SLE and in healthy controls. The bar represents the median value. Data were compared using the Mann-Whitney test; n.s. = not significant, **p < 0.01, ****p < 0.0001. Both values remained significant after Bonferroni correction. CXCL, chemokine (C-X-C motif) ligand; BLyS B, lymphocyte stimulator; IFNα, interferon alpha; PTX, pentraxin-related protein; MCP, monocyte chemotactic protein

A total of 96 patients with SLE and 13 healthy volunteers were recruited, of whom 95/96 (99.0%) and 12/13 (92.3%) were female, with a median (IQR) age of 50.7 (43.5, 58.8) and 40.0 (31.5, 48.5) years, respectively (Table 1). All healthy subjects were Caucasian and the patients with SLE were predominantly Caucasian (74/96, 77%) reflecting the patient population of Greater Manchester. The median disease duration was 11.7 (6.8, 21.3) years. All 10 cytokines were measurable in most patients. With the exception of IL-17, all cytokines were quantifiable in > 80% samples (see Additional file 1). Serum levels of CXCL10 and CXCL13 were significantly higher in patients with SLE compared to control (38.5 (19.9, 69.6) vs 11.8 (7.28, 16.9) pg/ml, *p* < 0.0001) and (351.6 (231.4, 568.2) vs. 234.7 (185.2, 239.2) pg/ml, *p* = 0.002), respectively (Fig. 1). Healthy subjects were younger, but in logistic regression models including age and gender, CXCL10 and CXCL13 remained significantly associated with the presence of SLE (OR 1.18 (1.06, 1.31), *p* = 0.002 and OR 1.01 (1.00, 1.01), *p* = 0.016). In an exploratory analysis, patients with SLE with arthritis (according to the ACR criteria) had increased levels of both CXCL10 and CXCL13. CXCL10 was also increased in patients with neurological involvement, and lower in patients with serositis (see Additional file 1).

**Table 1:** Characteristics of patients with SLE by disease activity status

	SLE (*n* = 96)	Healthy subject (*n* = 13)	*p* value
Age (years)	50.7 (43.9, 58.8)	40.0 (31.5, 48.5)	0.012
Gender (female)	95 (99.0%)	12 (92.3%)	0.626
Ethnicity			1.000
Caucasian	74 (77.1%)	13 (100%)	
Black Caribbean	6 (6.3%)		
Black African	3 (3.1%)		
Indian	2 (2.1%)		
Pakistani	3 (3.1%)		
Chinese	1 (1.0%)		
Other	3 (3.1%)		
Disease duration (years)	11.7 (6.84, 21.4)	–	–
≥ 4 ACR criteria	82 (85.4%)	–	–
ACR criteria			–
Malar rash	54 (56.3%)		
Discoid rash	16 (16.7%)		
Photosensitivity	60 (62.5%)		
Oral ulcers	54 (56.3%)		
Arthritis	62 (64.6%)		
Serositis	32 (33.3%)		
Renal	26 (27.1%)		
Neurological	10 (10.4%)		
Haematological	57 (59.4%)		
Immunological	48 (50.0%)		
ANA	82 (85.4%)		
Oral prednisolone use	41/93 (44.1%)		
Antimalarial use	66/93 (71.0%)		
Immunosuppressant use	32/92 (34.8%)		

Results show the number (percentage) or median (IQR) values. The Mann-Whitney U test or Fisher’s exact test was used to compare values in the active and the inactive groups

*SLE* systemic lupus erythematosus, *ACR* American College of Rheumatology, *ANA* antinuclear antibody

### Cytokine profiles and disease activity

**Fig. 2:**
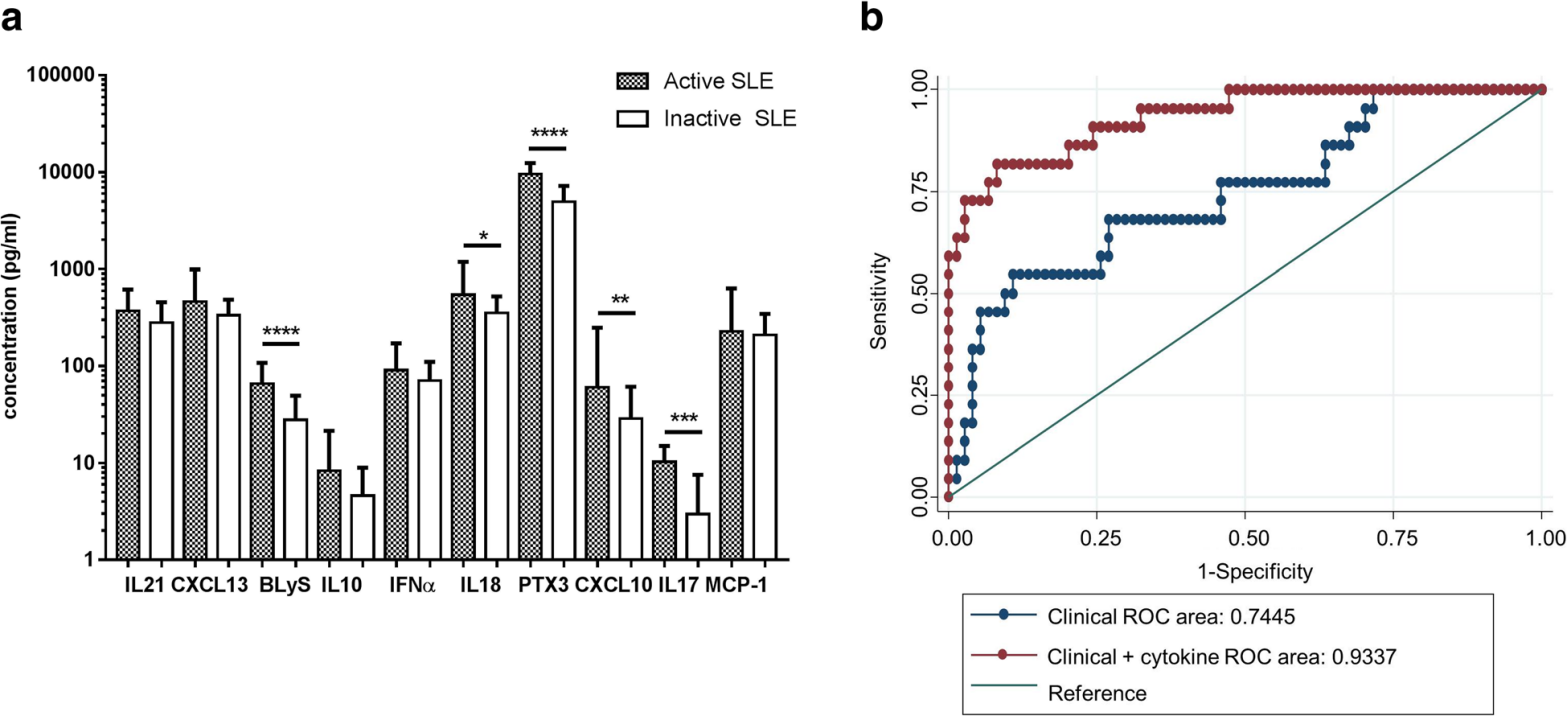
Area under the receiver operating characteristic (ROC) curve (AUC) for a multivariable logistic regression model of clinically active disease. **a** Cytokine levels in active and inactive systemic lupus erythematosus (SLE). The bars show the median level and the error bars represent the IQR. Data were compared using the Mann-Whitney U test: *p < 0.05, **p < 0.01, ***p < 0.001, ****p < 0.0001. After Bonferroni correction, IL-18 was no longer statistically significantly increased in patients with SLE. **b** The ROC curve is shown for the basic clinical model (blue line) and the same model with the addition of IL-10, IL-21, pentraxin (PTX)-3 and chemokine (C-X-C motif) ligand (CXCL)-10 (four-cytokine model) (red line). The AUC was 0.7312 and 0.9261, respectively (*p* < 0.001). BLyS B, lymphocyte stimulator; IFNα, interferon alpha; MCP, monocyte chemotactic protein

In the SLE patient group, 22/96 patients (23%) had active disease as defined above. The active group was younger (42.5 (31.0, 53.0) vs 52.5 (45.7, 60.7) years, *p* = 0.0016) and had a shorter duration of disease (7.16 (3.58, 15.0) vs 12.7 (8.11, 25.1) years, *p* = 0.0174) than patients with inactive SLE. The frequency of steroid prescription (19/22 (86.3%) vs 22/71 (31.0%), *p* < 0.0001) and the median daily dose (12.5 (7.5, 17.5) vs 7 (5, 10) mg, *p* = 0.002) was also greater in patients with active disease. There was no difference in the frequency of anti-malarial or immunosuppressant use between patients with active and inactive disease (*p* = 0.594 and *p* = 0.305, respectively). Serum levels of BLyS, IL-17, IL-18, CXCL10 and PTX3 were all significantly higher in patients with active disease (Fig. 2). In logistic regression models adjusted for age, gender, disease duration, ethnicity and concomitant steroid use, PTX3 and CXCL10 remained significantly associated with the presence of active disease (OR 1.23 (1.05, 1.45) per 1000 pg/ml and 1.02 (1.00, 1.03) per pg/ml) (Table 2). In a sensitivity analysis, addition of immunosuppressant use to the model did not change these associations (data not shown).

**Table 2:** Association between cytokine expression and presence of active disease in patients with SLE

	Unadjusted		Adjusted for age and gender		Fully adjusted^a^	
	OR (95% CI)	*p* value	OR (95% CI)	*p* value	OR (95% CI)	*p* value
IL-21^b^	1.06 (0.99, 1.13)	0.081	1.08 (0.99, 1.17)	0.077	1.06 (0.95, 1.18)	0.326
CXCL13^b^	1.07 (0.97, 1.18)	0.178	1.08 (0.97, 1.19)	0.177	1.05 (0.94, 1.18)	0.385
BLyS^b^	1.38 (0.96, 1.98)	0.079	1.58 (1.03, 2.42)	0.037	1.20 (0.82, 1.77)	0.350
IL-10	1.07 (1.01, 1.13)	0.013	1.08 (1.02, 1.14)	0.009	1.04 (0.99, 1.10)	0.143
IFNα^b^	1.27 (0.98, 1.64)	0.069	1.30 (0.99, 1.71)	0.056	1.14 (0.88, 1.48)	0.330
IL-18^b^	1.20 (1.05, 1.37)	0.009	1.25 (1.06, 1.47)	0.007	1.18 (0.98, 1.43)	0.075
PTX3^c^	1.33 (1.13, 1.56)	0.001	1.29 (1.11, 1.50)	0.001	1.23 (1.04, 1.46)	0.013
CXCL10	1.00 (1.00, 1.02)	0.022	1.01 (1.00, 1.02)	0.003	1.02 (1.00, 1.03)	0.008
IL-17	1.07 (1.01, 1.13)	0.020	1.06 (1.00, 1.13)	0.062	1.03 (0.98, 1.09)	0.279
MCP-1^b^	1.16 (1.00, 1.36)	0.056	1.20 (0.99, 1.45)	0.070	1.13 (0.90, 1.41)	0.294

Logistic regression models to show the odds of having active disease for every 1 pg/ml increase in the concentration of cytokine

*SLE* systemic lupus erythematosus, *OR* odds ratio, *95% CI* 95% confidence intervals, *CXCL* chemokine (C-X-C motif) ligand, *BLyS* B lymphocyte stimulator, *IFNα* interferon alpha, *PTX* pentraxin-related protein, *MCP* monocyte chemotactic protein

^a^Model adjusted for age, gender, disease duration, ethnicity (Caucasian vs non-Caucasian) and current steroid use

^b^Per 100 pg/ml increase

^c^Per 1000 pg/ml increase

Multivariable logistic regression models were used to determine whether measurement of these 10 cytokines could help to better identify patients with active disease, beyond the commonly used markers of low serum complement and raised anti-dsDNA. In a backwards stepwise logistic regression model, CXCL10, IL-10, IL-21 and PTX3 were all retained with *p* < 0.1.

The AUC of a basic multivariable model comprising age, ethnicity, disease duration, low serum complement and raised anti-dsDNA was 0.7445. The addition of the four cytokines (CXCL10, IL-10, IL-21 and PTX3) significantly increased the AUC to 0.9337 (*p* = 0.002) suggesting that measurement of these cytokines significantly improved the ability of the model to differentiate between patients with active and inactive disease (Fig. 2). There was no additional benefit of using all 10 cytokines in the model (data not shown).

### Cytokine clustering in patients with SLE

**Fig. 3:**
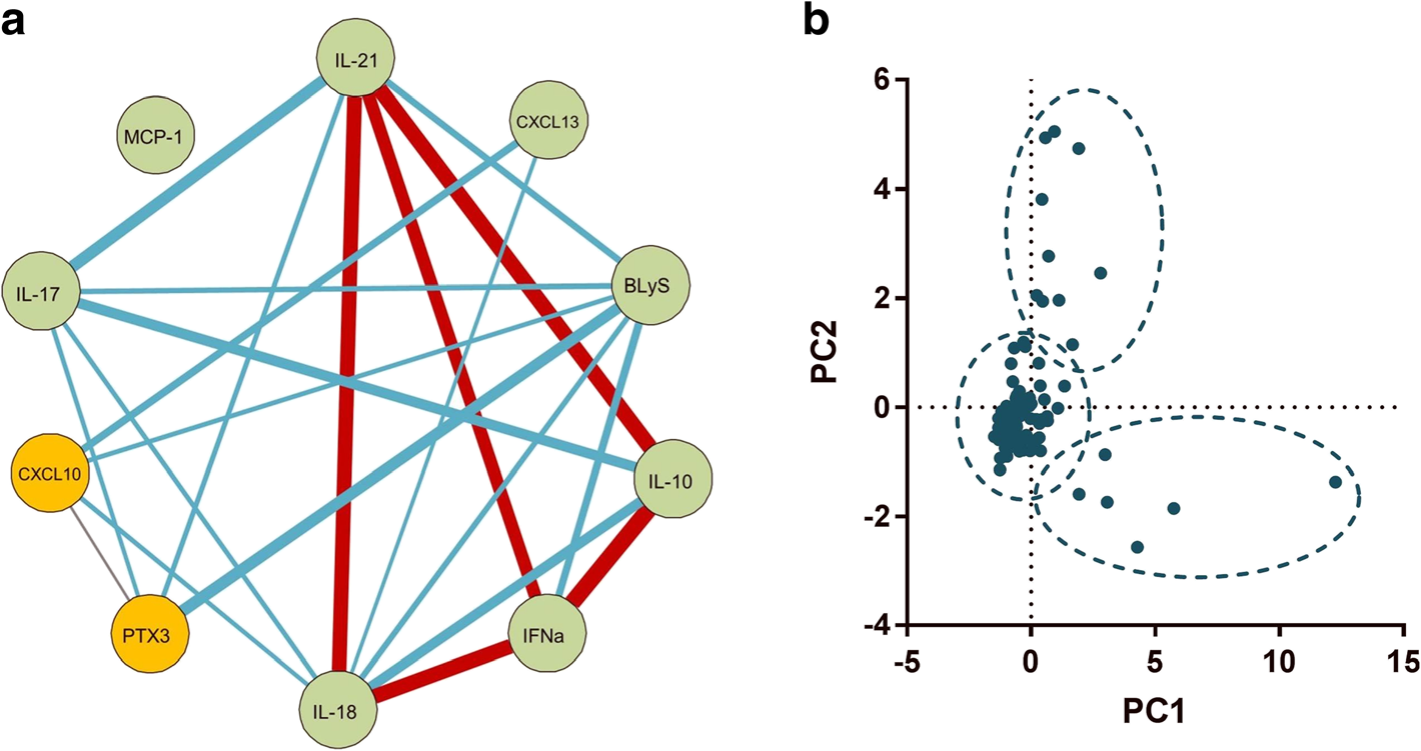
Cytokine expression and cluster analysis of patients with systemic lupus erythematosus. **a** Correlation between the 10 cytokines: the 2 cytokines associated with active disease are shown in gold. The lines represent correlation with a *p* value <0.05. The thickness and colour of the lines show the strength of the Spearman correlation: red, *r* > 0.4; blue, *r* 0.21–0.4; grey, *r* < 0.2. **b** Scatterplot of the first two principal components (PC1 and PC2) showing three main groups. CXCL, chemokine (C-X-C motif) ligand; BLyS B, lymphocyte stimulator; IFNα, interferon alpha; PTX, pentraxin-related protein; MCP, monocyte chemotactic protein

Immune/inflammatory mediators, including cytokines and chemokines, do not necessarily function in isolation from one another and therefore examination of cytokine/chemokine groups may be more informative than individual cytokines. A Spearman correlation matrix demonstrated that with the exception of MCP-1, each cytokine was significantly correlated (*p* < 0.05) with at least one other cytokine (Fig. 3). The strongest correlation was observed between IL-10 and IFNα (Spearman *r* = 0.464, *p* < 0.0001). There was only modest correlation between some cytokines that were increased in patients with active disease, for example for correlation between BLyS and CXCL10, Spearman *r* = 0.248 (*p* = 0.015), suggesting that active disease may be associated with more than one discrete pattern of cytokines.

Principal component analysis (PCA) of the 10 cytokines was performed in the patients with SLE to determine whether any unique groups of cytokines could be identified. Using the Kaiser criterion, three components were retained that had an Eigenvalue > 1. These three components described 57.9% of the variance in cytokine levels in our cohort. A scatterplot of the first two principal components are shown in Fig. 3. K-means clustering of the standardised levels of the 10 cytokines was then performed to cluster patients into k = 3 groups (Fig. 3). The 3 groups comprised 6, 11 and 79 patients, respectively. Groups 1 and 2 were formed principally of patients with active disease (4/6 (67%) and 8/11 (73%), respectively) whilst only 10/79 (12.7%) patients in group 3 had active disease (*p* < 0.0001). In keeping with this, oral corticosteroid use was higher in groups 1 and 2 compared to group 3 (*p* = 0.002), but there were no significant differences in antimalarial or other immunosuppressant use. In a sensitivity analysis including the healthy control samples all healthy subjects were clustered into group 3 (data not shown).

**Fig. 4:**
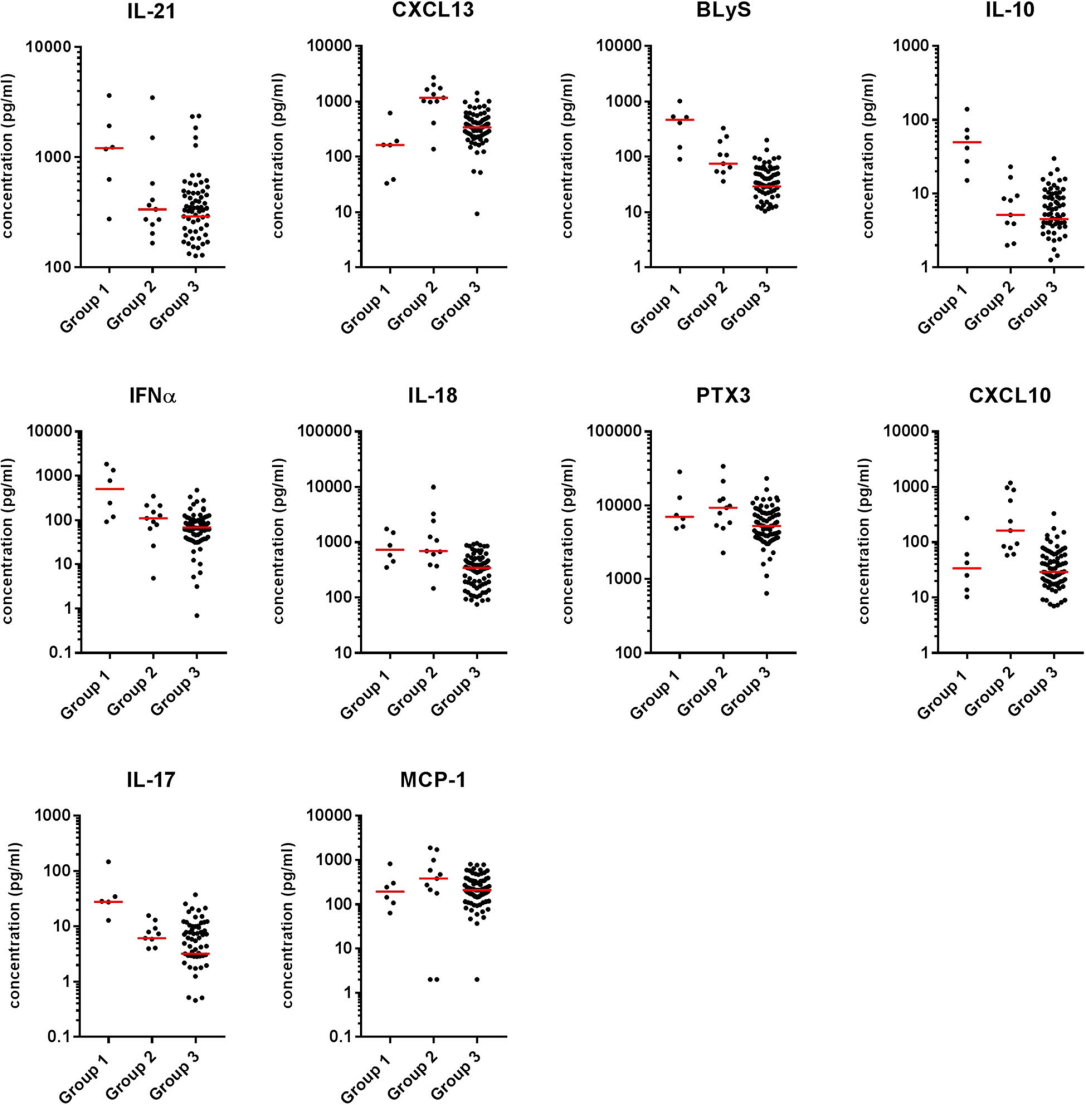
Cytokine levels in the 3 groups of patients with systemic lupus erythematosus (SLE). The serum levels of the 10 cytokines in the patients with SLE are shown according to the group allocation. Each dot represents a single patient and the bar shows the median value for the group. CXCL, chemokine (C-X-C motif) ligand; BLyS B, lymphocyte stimulator; IFNα, interferon alpha; PTX, pentraxin-related protein; MCP, monocyte chemotactic protein

In terms of the cytokines contributing to each cluster, IL-21, BLyS, IL-10, IFNα and IL-17 were increased in group 1 (median fold change between 3.6 and 9.6 compared to group 2). In contrast, CXCL10 and CXCL13 were highly expressed in group 2 (fold change compared to group 1, 4.8 and 7.2, respectively). IL-18 was strongly increased in both groups 1 and 2 and levels of PTX3 were moderately higher in group 2. All 10 cytokines were lower in group 3 than in groups 1 and 2. It was notable that levels of those cytokines that were increased in group 1 were much lower both in groups 2 and 3 (Fig. 4).

### Clinical features of clusters

The groups were similar in terms of age, ethnicity and disease duration (not shown). More patients in group 1 (3/6, 50%) were likely to be current smokers compared to groups 2 and 3 (0/11 and 6/79 (8%), respectively) (*p* = 0.001), but there was no difference in the frequency of those who ever smoked (*p* = 0.289). Patients with inflammatory arthritis (according to the updated 1997 ACR classification criteria for SLE) were significantly enriched in group 2 (11/11, 100%) compared to groups 1 (4/6, 67%) and 3 (47/79, 59%) (*p* = 0.031). There were no differences in the frequency of any of the other ACR classification criteria.

**Fig. 5:**
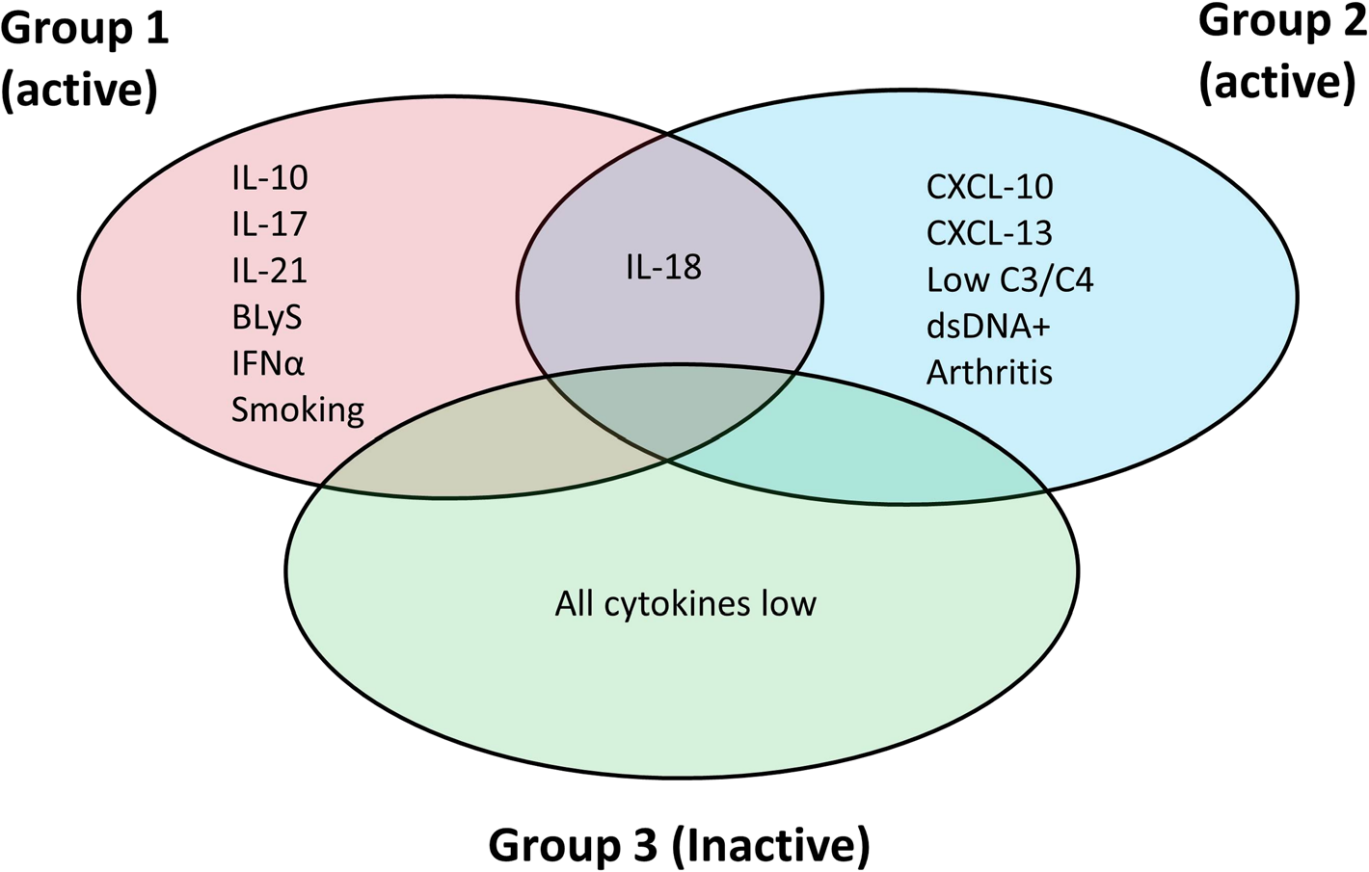
Summary of cytokine groups and significant clinical features. The diagram summarises the distribution of cytokines between the 3 groups in relation to the clinical features and serological markers. CXCL, chemokine (C-X-C motif) ligand; BLyS B, lymphocyte stimulator; IFNα, interferon alpha; PTX, pentraxin-related protein; MCP, monocyte chemotactic protein; dsDNA, double-stranded DNA

The 3 groups also differed in terms of serological markers. At the time of sampling, patients in group 2 were more likely to have a positive anti-dsDNA titre (*p* = 0.044) and lower levels of C3 (*p* = 0.003) and C4 (*p* = 0.034) complement. The cytokine, clinical and serological features of the 3 groups are summarised in Fig. 5.

### Stability in cytokine levels over time

Cytokine levels were measured at a second time point in a subgroup of clinically stable patients with SLE. The median (IQR) time between samples was 107 (91, 133) days. The intra-individual differences in cytokine levels between study visits were largest for MCP-1 (14.1%) and smallest for IFNα (− 1.35%). Cytokine levels only differed significantly between the two study visits for MCP-1 (see Additional file 1).

## Discussion

In this study cytokine profiles were identified in patients with SLE that were associated with the presence of active disease and with specific clinical features. The use of high-sensitivity quantification enabled detection of all 10 cytokines in the majority of patient samples, offering advantages over singleplex plate-based ELISA. Only a small number of samples had undetectable levels, which facilitated comparison of the levels of all 10 cytokines across the cohort. Both CXCL10 and CXCL13 were increased in patients with SLE compared to healthy subjects after adjustment for potential confounding factors. In a small case-control study by Bauer et al. (2006) a number of chemokines including CXCL10, CXCL13, CXCL9, MCP-1 (CCL2) and CCL8 were found to be increased in patients with SLE compared to controls [13]. This study compared patients with SLE on the basis of their IFN levels, which may therefore bias the observations towards IFN-related cytokines.

In this SLE cohort approximately 25% of patients had clinically active disease. As expected, those with active disease were younger, with shorter disease duration, and were receiving significantly higher doses of prednisolone whilst awaiting changes to their immunosuppressant therapy. Two cytokines, CXCL10 and PTX3, remained associated with active disease after adjustment for important confounding factors (including steroid use). Of these, CXCL10 had the strongest association with disease activity. The association between CXCL10 and active disease in SLE supports the results of two similar-sized studies [14, 15]. Interestingly, CXCL10/13 levels were not uniformly increased across the SLE cohort but appeared to be restricted to a subset of patients who were more likely to have serologically active disease (high anti-dsDNA and low complement) and inflammatory arthritis. Recently a small study by Ribeiro et al. (2018) identified that patients with SLE with Jaccoud’s arthropathy were significantly more likely to be ds-DNA positive and tended to have higher CXCL13 levels, although in this study there was no association between CXCL13 and synovitis or the arthritis component of the SLEDAI score [16]. CXCL13 has also been identified in the serum of patients with rheumatoid arthritis where it is associated with active disease [17].

PTX3 is an acute phase protein predominantly released by the innate immune system. In our study, PTX3 was significantly associated with disease activity both in isolation and as part of the four-cytokine model. Whilst this confirms the work of Assandri et al. [18], others have not found such an association [19].

In a combined model, the addition of four cytokines (IL-21, CXCL13, IL-10 and PTX3) significantly improved the ability of the model to predict the presence of active disease beyond the established markers of raised anti-dsDNA titre and low serum complement. These cytokines in combination were better predictors than any one alone (not shown). These cytokines may therefore have value as novel biomarkers for active SLE, although validation in further cohorts is needed.

The majority of the cytokines measured were significantly correlated with at least one other cytokine. A notable exception was MCP-1, which was not associated with any of the other nine cytokines. The importance of serum MCP-1 levels in SLE remains uncertain, as in a study by Zivkovic et al. (2017) urinary, but not serum levels were associated with the presence of lupus nephritis [20]. Cluster analysis identified three groups of patients, the largest of which had lower levels of all 10 cytokines and contained predominantly clinically inactive patients. The remaining two groups had mostly active disease and, with the exception of IL-18, had distinct cytokine profiles.

Group 1 had high levels of known lupus-related cytokines, including IFNα and BLyS, and pro-inflammatory cytokines including IL-17. Interestingly, group 1 patients were more likely to be smokers and the effect of smoking on the levels of these cytokines warrants further investigation. In contrast, group 2 contained increased levels of CXCL10 and CXCL13. In a study of 67 patients by Pacheco et al. (2017), using cytokine levels alone four groups were identified: neutral, chemokine, G-colony stimulating factor (G-CSF)-dominant and IFNα/pro-inflammatory [21]. Despite marked differences in the cytokines measures, our larger study also demonstrates “chemokine” and “cytokine” clusters of patients.

The clustering of BLyS in group 1 but low complement/high ds-DNA in group 2 was an unexpected finding. In the clinical trials of the anti-BLyS monoclonal antibody, belimumab, patients with low C3/4 and/or high anti-dsDNA were more likely to respond to BLyS inhibition [22]. The split of BLyS and these serological markers into different groups therefore requires validation and further study.

### Limitations of the study

Although this study identifies important associations between cytokines and disease in SLE, it has a number of limitations. First, cytokines were measured at either a single time point or at two time points, which may not adequately capture any fluctuations over time. Further studies are needed to determine how these individual cytokines and groups of cytokines change in response to disease flare/remission in patients with SLE. Second, the number of healthy subjects was relatively small, which may limit the ability to detect differences between healthy controls and patients with SLE, especially as we observed different cytokine patterns within the SLE group. The comparison between active and inactive SLE was also limited by modest numbers. Despite this, however, we identified a number of cytokines that were associated with active disease. Finally, the patients had established disease and some were receiving background treatment, which may affect cytokine levels. It is therefore not possible to comment on whether these findings are relevant to the early disease period.

## Conclusions

We have identified novel subsets of patients with active SLE on the basis of cytokine levels using a high-sensitivity bead array. These two groups have different clinical and serological features suggesting differences in pathogenesis. The number of subjects in our study was relatively small and so larger studies are required to confirm these findings and to more accurately classify patients on the basis of their immunophenotype.

## Additional file

Additional file 1: **Table S1.** Cytokine expression in patients with SLE measured using high-sensitivity bead array. **Table S2.** Cytokine levels over time in patients with stable SLE. **Table S3**. Association between SLE clinical features and CXCL10 and CXCL13 levels. (DOCX 9 kb)

## Abbreviations

ACR: American College of Rheumatology
BILAG: British Isles Lupus Assessment Group
BLyS: B lymphocyte stimulator
CXCL: Chemokine (C-X-C motif)
dsDNA: Double-stranded DNA
ELISA: Enzyme-linked immunosorbent assay
IFN: Interferon
IL: Interleukin
IQR: Interquartile range
MCP: Monocyte chemotactic protein
NHS: National Health Service
NIHR: National Institute for Health Research
PCA: Principal component analysis
PTX: Pentraxin-related protein
SLE: Systemic lupus erythematosus
SLEDAI-2K: Systemic lupus erythematosus disease activity index 2000
