# Supplementary material for: Cytokine profiling in active and quiescent SLE reveals distinct patient subpopulations
Source: Arthritis Res Ther. 2018 Aug 9;20:173. doi: 10.1186/s13075-018-1666-0 (PMC6085716; doi:10.1186/s13075-018-1666-0)
Supplement: Supplementary file 1 — Table S1. Cytokine expression in patients with SLE measured using high-sensitivity bead array. Table S2. Cytokine levels over time in patients with stable SLE. Table S3. Association between SLE clinical features and CXCL10 and CXCL13 levels. (DOCX 9 kb) [file 13075_2018_1666_MOESM1_ESM.docx]

Supplementary Data

Table S1: Cytokine expression in SLE patients measured using high sensitivity bead array

| Cytokine | Number (%) patients detectable levels (>0pg/ml) | Number (%) healthy subjects with detectable levels |
| --- | --- | --- |
| IL-21 | 85 (86) | 12 (92.3) |
| CXCL13 | 96 (100) | 13 (100) |
| BLyS | 79 (82) | 13 (100) |
| IL-10 | 79 (82) | 13 (100) |
| IFNα | 92 (96) | 13 (100) |
| IL-18 | 96 (100) | 13 (100) |
| PTX3 | 96 (100) | 13 (100) |
| CXCL10 | 96 (100) | 13 (100) |
| IL-17 | 70 (73) | 11 (84.6) |
| MCP-1 | 96 (100) | 13 (100) |

Table S2: Cytokine levels over time in patients with stable SLE

| Cytokine | Visit 1 | Visit 2 | Intra-subject difference | Median % change from baseline | Difference (visit 1 and visit 2) |
| --- | --- | --- | --- | --- | --- |
| IL-21 | 290.0 (78.9, 443.6) | 327.6 (94.9, 431.7) | 0 (-78.9, 64.3) | -13.4% | 0.6916 |
| CXCL13 | 316.2 (229.5, 422.8) | 286.5 (204.3, 456.6) | 5.94 (-56.2, 63.0) | 2.53% | 0.7770 |
| Blys | 33.7 (18.8, 53.8) | 36.4 (21.3, 69.6) | 7.51 (-8.25, 14.2) | 10.9% | 0.0854 |
| IL-10 | 4.05 (1.26, 8.81) | 3.94 (0.25, 8.13) | 0 (-2.10, 0.87) | -9.87% | 0.4292 |
| IFNα | 77.3 (41.4, 103.7) | 78.2 (45.7, 112.7) | 0 (-24.4, 13.5) | -1.35% | 0.7386 |
| IL-18 | 339.0 (162.4, 509.2) | 300.4 (146.8, 424.4) | -9.50 (-37.1, 52.5) | -4.18% | 0.9406 |
| Pentraxin 3 | 5058.2 (4056.5, 7331.8) | 6676.3 (4188.2, 8054.4) | 380.7 (-417.4, 1558.6) | 9.42% | 0.0567 |
| CXCL10 | 39.1 (17.7, 61.8) | 36.9 (22.7, 70.2) | 1.83 (-10.7, 9.4) | 4.70 | 0.8580 |
| IL-17 | 1.82 (0, 5.51) | 3.35 (0, 5.88) | 0 (0, 2.79) | 8.56% | 0.0859 |
| MCP-1 | 177.9 (146.0, 254.5) | 223.0 (150.6, 337.2) | 19.11 (-3.93, 76.7) | 14.1% | 0.0264 |

Values are expressed in pg/ml, and represent the median (IQR). Comparisons were made using the Wilcoxon matched-pairs signed rank test.

Table S3: Association between SLE clinical features and CXCL10 and CXCL13 levels

| Disease feature (as per ACR criteria) | CXCL10 (pg/ml) | | | CXCL13 (pg/ml) | | |
| --- | --- | --- | --- | --- | --- | --- |
|  | Clinical feature present | |  | Clinical feature present | |  |
|  | Yes | No | P | Yes | No | P |
| Malar rash  (yes= 54/96) | 28.7 (19.9, 64.5) | 40.1 (23.9, 70.7) | 0.4513 | 382 (296, 536) | 311 (201, 568) | 0.1357 |
| Discoid rash  (yes= 16/96) | 35.9 (19.6, 67.6) | 43.8 (21.9, 90.3) | 0.3924 | 348 (237, 568) | 362 (216, 747) | 0.9608 |
| Photosensitivity  (yes= 60/96) | 47.1 (22.3, 81.8) | 31.7 (18.6, 60.7) | 0.1661 | 416 (244, 627) | 327 (231, 487) | 0.1137 |
| Mucosal ulcers  (yes= 54/96) | 33.4 (17.7, 64.5) | 40.0 (23.3, 75.6) | 0.2774 | 329 (254, 537) | 369 (225, 581) | 0.8942 |
| Arthritis  (yes=62/96) | 27.7 (17.7, 43.4) | 44.3 (23.9, 79.0) | 0.0351* | 296 (213, 353) | 414 (275, 767) | 0.0019* |
| Serositis  (yes= 32/96) | 43.2 (21.6, 79.4) | 28.6 (19.6, 43.1) | 0.0373* | 341 (230, 578) | 364 (264, 568) | 0.7382 |
| Renal disease  (yes= 26/96) | 33.3 (19.2, 64.5) | 51.7 (21.3, 79.0) | 0.2838 | 342 (243, 581) | 359 (225, 555) | 0.7106 |
| Neurological disease  (yes= 10/96) | 33.7 (19.4, 64.5) | 66.9 (25.8, 273) | 0.0465* | 352 (243, 555) | 335 (163, 1030) | 0.8478 |

* statistically significant (compared using Mann-Witney U test)

Data shows the median (IQR) level for each cytokine.
